# Enhancing Electrochemical Non-Enzymatic Dopamine Sensing Based on Bimetallic Nickel/Cobalt Phosphide Nanosheets

**DOI:** 10.3390/mi15010105

**Published:** 2024-01-06

**Authors:** Zhi-Yuan Wang, Zong-Ying Tsai, Han-Wei Chang, Yu-Chen Tsai

**Affiliations:** 1 Department of Chemical Engineering, National Chung Hsing University, Taichung 402202, Taiwan; dog987784@gmail.com (Z.-Y.W.); g111065070@mail.nchu.edu.tw (Z.-Y.T.); 2 Department of Chemical Engineering, National United University, Miaoli 360302, Taiwan; 3 Pesticide Analysis Center, National United University, Miaoli 360302, Taiwan

**Keywords:** bimetallic nickel/cobalt phosphide nanosheets, phosphorization, synergistic effect of Ni/Co atoms, electrochemical non-enzymatic DA sensing

## Abstract

In this study, the successful synthesis of bimetallic nickel/cobalt phosphide nanosheets (Ni-Co-P NSs) via the hydrothermal method and the subsequent high-temperature phosphorization process were both confirmed. Ni-Co-P NSs exhibited excellent electrocatalytic activity for the electrochemical non-enzymatic DA sensing. The surface morphologies and physicochemical properties of Ni-Co-P NSs were characterized by atomic force microscopy (AFM), field-emission scanning (FESEM), field-emission transmission electron microscopy (FETEM), and X-ray diffraction (XRD). Further, the electrochemical performance was evaluated by cyclic voltammetry (CV) and differential pulse voltammetry (DPV). The metallic nature of phosphide and the synergistic effect of Ni/Co atoms in Ni-Co-P NSs provided abundant catalytic active sites for the electrochemical redox reaction of DA, which exhibited a remarkable consequence with a wide linear range from 0.3~50 μM, a high sensitivity of 2.033 µA µM^−1^ cm^−2^, a low limit of detection of 0.016 µM, and anti-interference ability. As a result, the proposed Ni-Co-P NSs can be considered an ideal electrode material for the electrochemical non-enzymatic DA sensing.

## 1. Introduction

Dopamine (DA) is a vital catecholamine neurotransmitter in the central nervous system of mammals. It primarily regulates human desire and transmits information related to excitement and happiness, thereby influencing human emotions [1]. The secretion and expression of DA occurs within highly specific regions of the human brain, including the ventral tegmental area (VTA) of the midbrain, the substantia nigra pars compacta, and the hypothalamic arcuate nucleus. Dopaminergic neurons located in the VTA are believed to mediate natural motivation, reward prediction, and contextual learning [2,3]. Dopaminergic neurons in the substantia nigra pars compacta play a vital role in motor symptoms, with their depletion leading to the characteristic motor dysfunction seen in Parkinson’s disease [4,5]. Additionally, dopaminergic neurons in the hypothalamic arcuate nucleus regulate the inhibition of the synthesis and secretion of prolactin, a protein hormone involved in prolactin homeostasis of the body [6,7]. Clearly, it is urgent to develop an accurate and efficient method for promptly detecting abnormal concentration levels of DA, averting potential social, psychological, and economic burdens on individuals and society. Several analytical methods have been developed to evaluate the concentration levels of DA, such as fluorescence [8], colourimetric assays [9], electrochemiluminescence [10], ultra-performance liquid chromatography-tandem mass spectrometry (UPLC/MS) [11], and electrochemistry [12,13,14]. Among these methods, electrochemical approaches stand out as powerful and widely used tools due to their low cost, rapid analysis time, immediate response, and ease of operation [15,16]. Electrochemical methods can be broadly classified into two major sensing platforms: enzymatic and non-enzymatic. Enzymatic electrochemical sensing offers advantages like high sensitivity and specificity. However, constructing enzymatic electrochemical sensing platforms involves interactions between enzymes and electrodes, which present limitations such as high cost, complex fabrication, poor reproducibility, and susceptibility to environmental factors like pH and temperature [17,18]. The development of electrochemical non-enzymatic sensing platforms allows for overcoming the limitations associated with current electrochemical enzymatic sensing. These advancements address the issues related to enzymes mentioned above.

The choice of appropriate electrode materials plays a pivotal role in constructing an electrochemical non-enzymatic sensing platform, determining the performance of electrochemical sensing. Previous reports suggest that an electrochemical sensing platform comprising transition metal compounds (TMCs), such as transition metal oxides (TMOs) [19], sulphides (TMSs) [20], nitrides (TMNs) [21], and phosphides (TMPs) [22,23] could offer universal design strategies for detecting DA electrochemically.

The properties of TMPs have drawn significant attention due to their natural abundance, high conductivity, electrocatalytic activity, and favourable physicochemical characteristics [24]. Among these compounds, the relatively low electronegativity of phosphorus (P) (2.19), compared to sulphur (S) (2.58), nitrogen (N) (3.04), and oxygen (O) (3.44) promotes a strong covalent bond between the transition metal and P atom. The relatively strong metal–ligand covalence leads to a weaker attraction to the electrons in the third orbitals of the transition metal atoms, which promotes excellent reaction kinetics for enhanced electrochemical performance, allowing outstanding electrocatalytic performance for promising applications in water splitting [25], supercapacitors [26], CO_2_ reduction [27], and battery [28]. When the transition metal elements increase to binary transition metal phosphides, it exhibits multiple oxidation states, fostering strong synergistic effects that enhance electrochemical performance [29,30,31]. Previous studies have highlighted the adjustable morphologies controlled by experimental synthetic factors, influencing the construction of 2D/3D hierarchical structures that offer increased active sites and efficient electron/carrier transfer, achieving enhanced electrochemical performance [32,33].

In this study, bimetallic nickel/cobalt phosphide nanosheets (Ni-Co-P NSs) were successfully synthesised using a facile hydrothermal method and subsequent the high-temperature phosphorization treatment. Ni-Co-P NSs exhibited a 2D structural properties and demonstrated excellent electrocatalytic performance compared to monometallic transition metal phosphides (NiP and CoP). The unique characteristics of the Ni-Co-P NSs, attributed to the metallic nature of phosphide and the synergistic effect of Ni/Co atoms, make them a promising electrode material for electrochemical non-enzymatic DA sensing.

## 2. Materials and Methods

### 2.1. Reagents

Cobalt (II) sulphate heptahydrate (CoSO_4_·7H_2_O), Nickel (II) sulphate hexahydrate (NiSO_4_·6H_2_O), and sodium phosphate monobasic monohydrate (NaH_2_PO_4_·H_2_O) were obtained by Alfa Aesar (Ward Hill, MA, USA). Glycerol and anhydrous ethanol (C_2_H_5_OH, 99.9%) were purchased from J.T. Baker (Phillipsburg, NJ, USA). Urea, Nafion^®^ solution (5 wt % in mixture of lower aliphatic alcohols and water), dopamine hydrochloride (DA), uric acid (UA), and L-Ascorbic acid (AA) were purchased from Sigma-Aldrich (St. Louis, MO, USA). The deionized water (DI water) was produced from the Milli-Q water purification system of Millipore Co. (Bedford, MA, USA). All chemicals were analytical grade and were used as received without further purification.

### 2.2. Synthesis of Nickel/Cobalt Phosphide Nanosheets (Ni-Co-P NSs)

**Figure 1:**
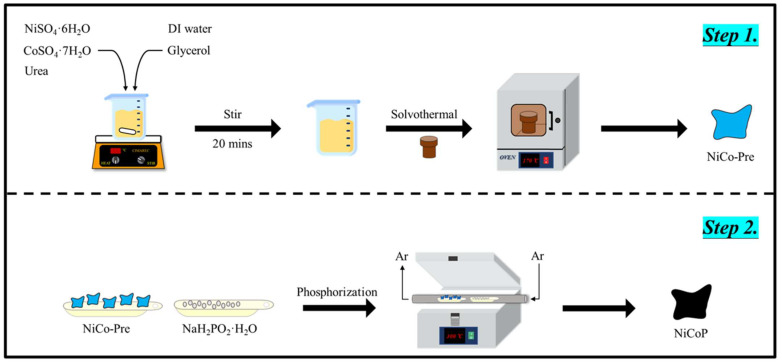
The schematic diagram for the synthesis of Ni-Co-P NSs.

The Ni-Co-P NSs were synthesised via a facile hydrothermal method and subsequent high-temperature phosphorization treatment. Typically, 1.5 mmol NiSO_4_·6H_2_O, 3 mmol CoSO_4_·7H_2_O, and 0.15 g urea were fully dissolved into a solution containing 50 mL DI water and 10 mL glycerol (*v*/*v* = 5:1). By stirring 20 min, hydrothermal process was carried out at 170 °C in a Teflon-lined stainless autoclave (100 mL) in an oven for 20 h. The resulting product was purified by repeated centrifugation and washing with DI water and anhydrous alcohol three times, and then dried in an oven at 70 °C overnight to obtain the Ni-Co precursor for subsequent phosphorization treatment. The subsequent the high-temperature phosphorization process was conducted via thermal treatment in a tube furnace by using NaH_2_PO_2_·H_2_O. The 40.0 mg obtained Ni-Co-Pre and 120.5 mg NaH_2_PO_2_·H_2_O were weighed and spread on two different ceramic boats, respectively, which were situated side by side at the centre of a tube furnace. The boat containing NaH_2_PO_2_·H_2_O was placed in the quartz tube at the upstream side of the tube furnace and another boat containing NiCo-Pre was placed at the downstream side of the tube furnace. Subsequently, these samples in the tube furnace were calcined to 300 °C for 2 h in the Ar atmosphere with a heating rate of 2 °C min^−1^. Then, Ni-Co-P NSs were obtained after naturally cooling to room temperature. The collected Ni-Co-P NSs was ready for subsequent characterization. The schematic diagram of the synthesis of Ni-Co-P NSs is shown in Figure 1. Moreover, to demonstrate Ni-Co-P NSs have a better catalytic performance than monometallic Ni and Co phosphides, monometallic Ni phosphide can be obtained without adding Co source (denoted as Ni-P) and monometallic Co phosphide can be obtained without Ni source (denoted as Co-P).

### 2.3. Fabrication of Ni-Co-P NSs Electrode

The Ni-Co-P NSs electrode was prepared by drop casting of Ni-Co-P NSs suspensions on the surface of the cleaned glassy carbon electrode (GCE, diameter 3 mm, Tokai Carbon, Tokyo, Japan). First, the bare GCE was rigorously polished with 0.3 and 0.05 μm alumina slurry, respectively, and cleaned with DI water and then dried at 70 °C in an oven for 20 min, which was used for further modifications. For the fabrication of Ni-Co-P NSs electrode, first, 2 mg of Ni-Co-P NSs was weighed and dispersed in 1 mL of 0.5 wt % Nafion^®^ solution via ultrasonic treatment for 30 min to form a homogeneous suspension. Then, suspension (6 μL) was dropping cast on cleaned GCE and dried in the oven as a working electrode (to be subsequently denoted as Ni-Co-P NSs/Nafion/GCE) for the following electrochemical measurement. For comparison, monometallic Ni and Co phosphides (Ni-P and Co-P) modified GCE were fabricated using the same method (denoted as Ni-P/Nafion/GCE and Co-P/Nafion/GCE).

### 2.4. Characterizations

The morphology was characterized by using the atomic force microscope (AFM, SPA-400, Hitachi, Tokyo, Japan), field-emission scanning electron microscopy (FESEM, JSM-7410F, JEOL, Akishima, Japan), and field-emission transmission electron microscopy (FETEM, JEM-2100F, JEOL, Akishima, Japan). The crystal phase was characterized by using the X-ray diffraction (XRD) (D8 Discover X-ray diffractometer with Cu Kα radiation (Bruker, Karlsruhe, Germany)). Electrochemical measurements were performed by using a three-electrode system composed of as-prepared Ni-Co-P NSs working electrode, a platinum wire counter electrode, and an Ag/AgCl (3 M KCl) reference electrode by an electrochemical analyser (Autolab, model PGSTAT30, Eco Chemie, Utrecht, The Netherlands). All electrochemical measurements were conducted in 0.1 M phosphate-buffered saline (PBS) as the supporting electrolyte in the absence and presence of DA at ambient temperature. Cyclic voltammetry (CV) curves and differential pulse voltammetry (DPV) curves were performed between 0~0.8 V.

## 3. Results and Discussion

**Figure 2:**
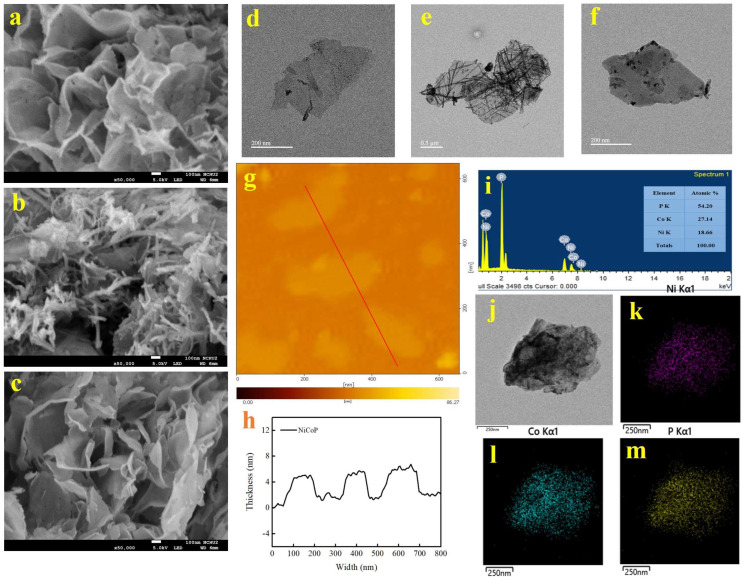
(**a**–**c**) FESEM and (**d**–**f**) FETEM images of NiP, CoP, and Ni-Co-P NSs. (**g**) AFM and (**h**) AFM cross section image of Ni-Co-P NSs. (**i**) EDS spectrum of Ni-Co-P NSs. (**j**) STEM images of Ni-Co-P NSs and corresponding EDS mapping images for (**k**) Ni, (**l**) Co, and (**m**) P elements.

The morphologies of NiP, CoP, and Ni-Co-P NSs were characterised using FESEM, FETEM, and AFM. FESEM and FETEM images of NiP, CoP, and Ni-Co-P NSs are shown in Figure 2a–f. The FESEM images showcase a highly interconnected network of NiP, CoP, and Ni-Co-P NSs. It can be observed that NiP displayed a nanosheets structure and CoP displayed a nanosheet-nanowire structure. And the Ni-Co-P NSs also displayed a mixture structure of 1D nanowires and 2D nanosheets, the 1D nanowires content in Ni-Co-P NSs was much less than that of the 1D nanowires content in CoP, which were well matched with those of the FETEM results. The mixture structure of 1D nanowires and 2D nanosheets were intercrossed to construct the 3D well-interconnected networks, suppling more abundant specific surface areas and active sites. This interconnected network structure is expected to facilitate rapid electron and carrier transfer during electrochemical processes, potentially leading to improved electrochemical responses. The AFM image (Figure 2g) and the corresponding cross-section (Figure 2h), taken along the red solid line of the Ni-Co-P NSs, confirm a consistent nanosheets (NSs) structure with an average thickness of approximately 4 nm and an average width of 200 nm, which significantly augment the electrode/electrolyte contact area, thereby promoting favourable electrochemical reaction kinetics. The surface elements composition and distribution of Ni-Co-P NSs were analysed using EDS (Figure 2i) and STEM analysis (Figure 2j) corroborated the well-distributed presence of Ni (Figure 2k), Co (Figure 2l), and P (Figure 2m) elements across the surface of the Ni-Co-P NSs. These findings establish the interconnected network consisting of nanosheet-nanowire structure and the uniform distribution of Ni, Co, and P elements in Ni-Co-P NSs, crucial for their electrochemical performance.

**Figure 3:**
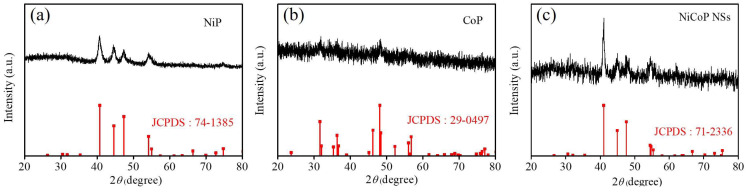
XRD patterns of (**a**) NiP, (**b**) CoP, and (**c**) Ni-Co-P NSs.

The XRD patterns of NiP, CoP, and Ni-Co-P NSs were compared with the standard patterns of NiP (JCPDS No. 74-1385), CoP (JCPDS No. 29-0497), and NiCoP (JCPDS No. 71-2336) [34] to characterize their structure and phase composition, as depicted in Figure 3. All the observed diffraction peaks in NiP, CoP, and Ni-Co-P NSs match well with the standard patterns, further confirming the successful synthesis of NiP, CoP, and Ni-Co-P NSs. In addition, it can be seen that the XRD patterns of NiP and NiCoP peaks were much sharper than that of CoP, indicating that the NiP and NiCoP have a higher degree of crystalline. In the XRD pattern of Ni-Co-P NSs, the most pronounced diffraction peak centred at 41.0° corresponds to the (111) plane of hexagonal NiCoP. Additionally, five relatively weaker diffraction peaks at 44.9°, 47.6°, 54.4°, 54.7°, and 55.3° can be attributed to the (201), (210), (300), (002), and (210) planes of hexagonal NiCoP, indicating the presence of smaller crystallite sizes and lower crystallinity. This outcome validates the successful synthesis of the 2D structure of Ni-Co-P NSs and confirms the formation of NiCoP in the hexagonal phase.

**Figure 4:**
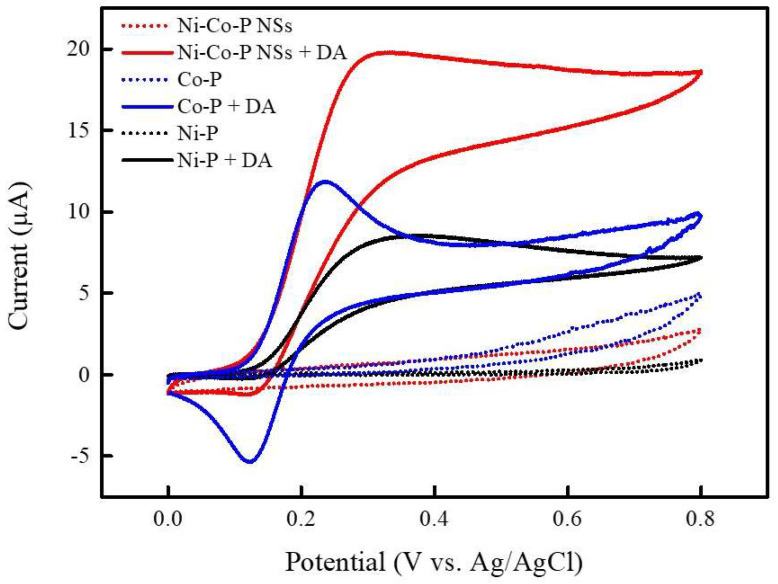
CV curves of Ni-P/Nafion/GCE (black line), Co-P/Nafion/GCE (blue line), and Ni-Co-P NSs/Nafion/GCE (red line) in the absence (dotted lines) and presence (solid lines) of 0.1 mM DA in 0.1 M PBS (pH 7.0) at a scan rate of 50 mV s^−1^.

**Figure 5:**
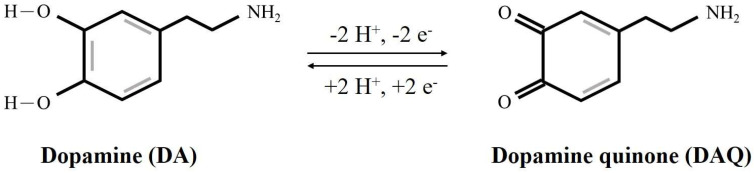
The electrochemical reaction mechanism of DA.

To assess the electrochemical properties for DA sensing, cyclic voltammetry (CV) was conducted on bimetallic nickel/cobalt phosphide nanosheets (Ni-Co-P NSs), monometallic Ni phosphides (Ni-P), and monometallic Co phosphides (Co-P). Figure 4 illustrates the CV curves of Ni-Co-P NSs/Nafion/GCE, Ni-P/Nafion/GCE, and Co-P/Nafion/GCE in 0.1 M PBS (pH 7.0) in the absence (dotted lines) and presence (solid lines) of 0.1 mM DA, using a scan rate of 50 mV s^−1^ within a potential window of 0–0.8 V. In the absence of DA, no oxidation or reduction peaks were observed across these electrodes. However, upon the addition of 0.1 mM DA, well-defined redox peaks corresponding to the electrochemical mechanism of DA emerged prominently at 0.12 and 0.31 V (vs. Ag/AgCl) for Ni-Co-P NSs/Nafion/GCE. Figure 5 presents the electrochemical redox mechanism of DA, involving a two-proton and two-electron transfer process, signifying the conversion between DA and dopamine quinone (DAQ). At a typical physiological pH, DA is positively charged (pKa 8.87), under oxidative conditions, DA undergoes deprotonation (DA → DAQ), resulting in a relatively higher negative charge. Simultaneously, the P atoms, with their lone pair of electrons, readily interact with H^+^ ions, becoming highly dense and positively charged. The strong electrostatic interactions between the DA derivatives and transition metal phosphides promote the electrochemical redox reaction of DA. However, the weak reduction peak of DAQ to DA under reductive conditions might be attributed to the strong adsorption of DAQ on the phosphides or the sluggish electron transfer between the electrode/electrolyte interface [35,36,37]. As observed in Figure 4, the CV curve of Ni-Co-P NSs/Nafion/GCE exhibited the most significant anodic current response to 0.1 mM DA compared to Co-P/Nafion/GCE and Ni-P/Nafion/GCE. These comparative results further validate that Ni-Co-P NSs/Nafion/GCE demonstrate a synergistic effect attributed to the metallic nature of phosphide and Ni/Co atoms in bimetallic Ni-Co phosphides. This synergy encompasses abundant catalytic active redox sites from Ni and Co metals, along with the relatively low electronegativity of P, enhancing a high degree of covalency in the Ni/Co-P bonding that can enables the fabricated Ni-Co-P NSs/Nafion/GCE to show excellent electrochemical performance.

**Figure 6:**
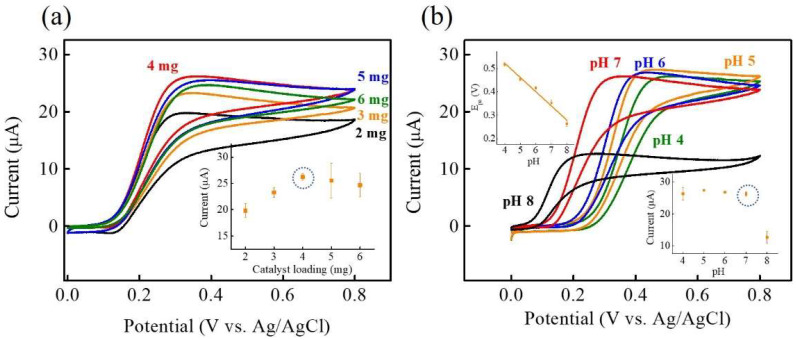
CV curves of Ni-Co-P NSs/Nafion/GCE at different (**a**) mass loading of Ni-Co-P NSs (2, 3, 4, 5, and 6 mg) and (**b**) pH (4, 5, 6, 7, and 8) in 0.1 M PBS in the presence of 0.1 mM DA at a scan rate of 50 mV s^−1^. Inset of (**a**) plot of the anodic peak current (I_pa_) versus the different mass loading of Ni-Co-P NSs. Insets of (**b**) plot of the anodic peak potential (E_pa_) versus pH (upper-left inset) and plot of the anodic peak current (I_pa_) versus pH (bottom-right inset). (The error bars represent the standard deviation of 3 repeat measurements).

The optimization of electrochemical DA sensing performance involved careful adjustments in both the mass loading of Ni-Co-P NSs and pH levels to achieve optimal results. In Figure 6a, the influence of Ni-Co-P NSs’ mass loading on anodic peak currents was explored. Ni-Co-P NSs/Nafion/GCE at different mass loadings of Ni-Co-P NSs were controlled by weighing 2–6 mg of Ni-Co-P NSs in 1 mL of 0.5 wt % Nafion to form a homogenous dispersion, and sequentially drop-casting the homogenous dispersion onto the GCE. Figure 6a shows the CV curves of Ni-Co-P NSs at different mass loadings of Ni-Co-P NSs in 0.1 M PBS (pH 7.0) in the presence of 0.1 mM DA at a scan rate of 50 mV s^−1^. The inset of Figure 6a shows the plot of the anodic peak currents versus mass loading of the Ni-Co-P NSs. As can be seen, the anodic peak currents increase when the loading of Ni-Co-P NSs is increased from 2 to 4 mg. However, when the loading of Ni-Co-P NSs exceeds 4 mg, the anodic peak currents gradually decrease due to the mass transfer limitation by excess mass loading [38]. Thus, 4 mg was chosen as the optimised mass loading of Ni-Co-P NSs for the following experiments. Figure 6b shows the CV curves of Ni-Co-P NSs at different pHs (from pH 4 to 8) in the presence of 0.1 mM DA. The anodic peak potential shifted negatively with increasing pH values, indicating the involvement of proton/electron transfer in the electrochemical redox reactions of DA [39], as described in Figure 5. The corresponding linear relationship between anodic peak potential (E_pa_) and pH was calculated as follows: E_pa_ (V) = 0.77 − 0.06 pH (R^2^ = 0.97463) (see the top left inset of Figure 6b). Furthermore, the slope of 0.06 was close to the theoretical Nernstian value of 0.059 V/pH referring to the electrochemical redox mechanism of DA involving two proton/electron transfer processes [13,40]. Notably, the change of the anodic peak current was not evident from pH 4.0 to 7.0, and the sensing current dramatically decreased with the subsequent increase of pH from 7.0 to 8.0. Moreover, the bottom-right inset of Figure 6b shows the plot of the anodic peak currents versus pH. The best anodic peak current was found at pH 7.0, which works well in physiological pH conditions to promote its practical use in electrochemical sensing devices [41]. Based on the above-mentioned optimal experiments, 4 mg mass loading of Ni-Co-P NSs and pH 7.0 (marked by the dashed circle in inset of figure) were chosen as optimal parameters to further improve the electrochemical response towards DA sensing.

**Figure 7:**
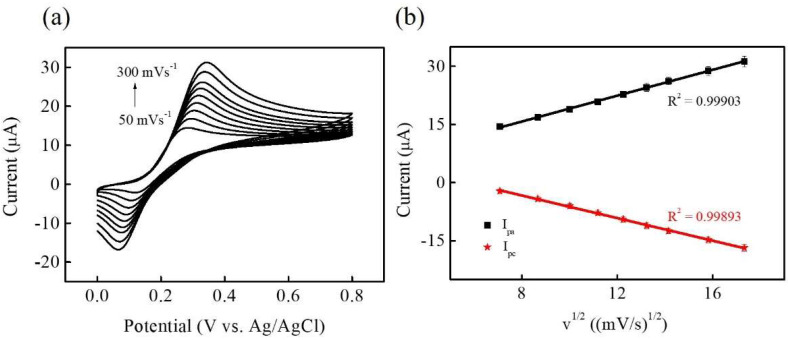
(**a**) CV curves of Ni-Co-P NSs/Nafion/GCE in 0.1 M PBS (pH 7.0) in the presence of 0.1 mM DA at different scan rates from 50 to 300 mVs^−1^. (**b**) The plot of the anodic peak current (I_pa_) and the cathodic peak current (I_pc_) versus square root of scan rate (*v*^1/2^).

Figure 7a displays the CV curves of Ni-Co-P NSs/Nafion/GCE in 0.1 M PBS (pH 7) in the presence of 0.1 mM DA at varying scan rates from 50 to 300 mVs^−1^. The redox peak current increased with increasing scan rates. In Figure 7b, the relationship between anodic and cathodic peak current (I_pa_ and I_pc_) against the square root of the scan rates (*v*^1/2^) within the range of 50–300 mVs^−1^ is depicted. It demonstrates a linear relationship between both anodic and cathodic peak currents (I_pa_ and I_pc_) and the square root of the scan rate. The linear equations can be expressed as I_pa_ (μA) = 2.4316 + 1.6658 *v*^1/2^ ((mVs^−1^)^1/2^) (R^2^ = 0.99903) and I_pc_ (μA) = 8.3246 − 1.4558 *v*^1/2^ ((mVs^−1^)^1/2^) (R^2^ = 0.99893), indicating that the electrochemical redox behaviour of DA sensing is attributed to diffusion processes [42].

**Figure 8:**
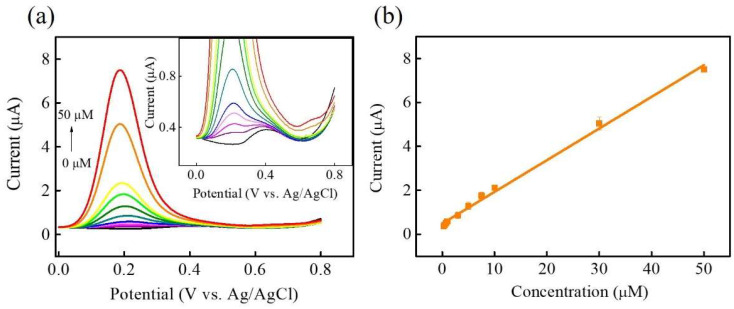
(**a**) DPV responses of Ni-Co-P NSs/Nafion/GCE in 0.1 M PBS (pH 7.0) with the different DA concentrations ranging from 0.3 to 50 μM. (**b**) The calibration curve of the DA sensor. Inset of (**a**) DPV curves of NiCoP/Nafion/GCE in 0.1 M PBS (pH 7.0) at low concentration region.

Under optimal parameters, the electrochemical performance of Ni-Co-P NSs/Nafion/GCE for the electrooxidation of DA sensing was analysed using differential pulse voltammetry (DPV) in 0.1 M PBS (pH 7) with various DA concentrations (0–50 μM) added to evaluate the feasibility of the fabricated electrochemical sensor. The experimental parameters for the DPV analysis technique were potential window = 0–0.8 V, scan rate = 20 mVs^−1^, modulation time = 0.05 s, internal time = 0.2 s, and step potential = 0.004 V. Figure 8a shows the DPV response at Ni-Co-P NSs/Nafion/GCE with increasing DA concentrations from 0 to 50 μM. It is evident that the DPV response increased with the increase in DA concentration, and the inset of Figure 8a shows an amplified view of the DPV responses in the low-concentration region. The DPV responses within the concentration range of 0 to 50 μM were recorded to obtain the corresponding calibration plot, displayed in Figure 8b. The linear regression equation between anodic peak current (I_pa_) versus the concentrations of DA (Conc.) can be expressed as I_pa_ (μA) = 0.19038 + 0.14516 Conc. (μM). The DA calibration curve was highly linear from 0.3 to 50 μM (R^2^ = 0.99509). The sensitivity, limit of detection (LOD) based on 3 S_b_/m, and limit of quantification (LOQ) based on 10 S_b_/m (S_b_ is the standard deviation of the blank signals for *n* = 3, and m is the slope of the calibration plot) are estimated as 2.033 µA µM^−1^ cm^−2^, 0.016 µM, and 0.053 mM, respectively. The proposed Ni-Co-P NSs/Nafion/GCE showed good comparability with some previous reports concerning electrochemical non-enzymatic DA sensors based on different transition-metal (including metal Ni, Co, or Ni/Co) compound materials (Table 1) [39,43,44,45,46,47].

**Figure 9:**
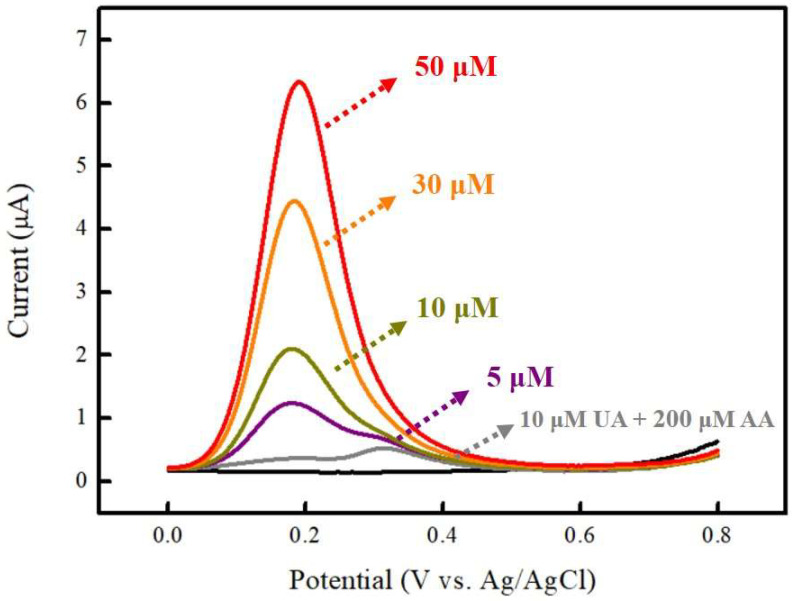
Interference test of Ni-Co-P NSs/Nafion/GCE in 0.1 M PBS (pH 7.0) in the presence of 10 μM UA and 200 μM AA by adding different concentrations of DA (5, 10, 30, and 50 μM). Black line is 0.1 M blank PBS.

For electrochemical non-enzymatic DA sensing, anti-interference is an essential parameter to evaluate DA sensing performance in practical applications. In the physiological environment, two common biomolecules, uric acid (UA) and ascorbic acid (AA), often coexist with DA in human body fluids, and their oxidation potentials overlap on conventional bare electrodes [48]. The proposed Ni-Co-P NSs/Nafion/GCE showed outstanding electrocatalytic activity, which has been demonstrated to overcome signal interferences from UA and AA with DA. According to previous reports [49,50], the UA concentration in human blood plasma is controlled in the range of 140–420 μM, while in human cerebrospinal fluid, it is about 10 times lower due to limited transport pathways across the blood–brain barrier, protecting the brain from blood. In addition, the AA concentration in the human body ranges from about 200–500 μM. Figure 9 shows the interference test of Ni-Co-P NSs/Nafion/GCE in 0.1 M PBS (pH 7.0) in the presence of 10 μM UA and 200 μM AA (covering normal levels in the human body) by adding different concentrations of DA (5, 10, 30, and 50 μM). The DPV response of DA increased with increasing DA concentrations in the presence of both UA and AA simultaneously. The response current of UA and AA exhibited much weaker signals. By linear regression analysis, the relationship between anodic peak current (I_pa_) versus the concentrations of DA (Conc.) in the presence of interfering substances can be expressed as I_pa_ (μA) = 0.21715 + 0.12436 Conc. (μM) (R^2^ = 0.99094). Compared to the slope and intercept of linear regression in the absence (in Figure 8) and presence (in Figure 9) of interfering substances with the determination of DA, there were no significant difference between the linear regression correlation slopes and intercepts, indicating that UA and AA do not interfere during DA sensing. Therefore, the interference from UA and AA can be disregarded. The proposed Ni-Co-P NSs/Nafion/GCE with excellent selectivity holds great promise for practical electrochemical DA sensing.

**Figure 10:**
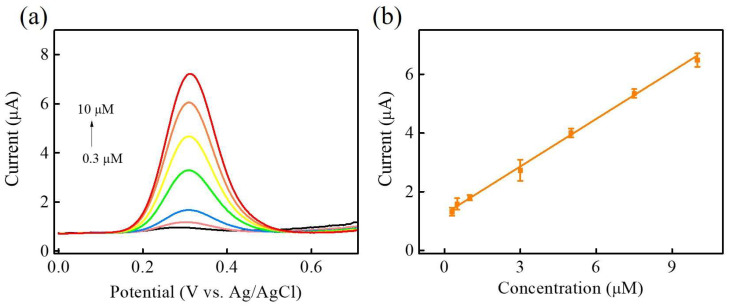
(**a**) DPV responses of Ni-Co-P NSs/Nafion/GCE in human serum samples with the gradual additions of dopamine concentration up to 10 μM. (**b**) Corresponding calibration plot.

To evaluate the practical applications of the proposed Ni-Co-P NSs/Nafion/GCE for dopamine sensing, the electrodes were subjected to DPV responses by determining DA in human serum. The human serum (from human male AB plasma, H4522) was purchased from Sigma-Aldrich (St. Louis, MO, USA) and the various DA concentrations in human serum samples were prepared by mixing 0.1 M PBS (pH 7.0) and human serum with the gradual additions of DA concentration up to 10 μM (the human serum samples were diluted 5 times with PBS). It can be seen that the DPV responses increased linearly from human serum samples increasing DA concentrations (Figure 10a). The corresponding calibration plot of DPV responses against DA concentrations was displayed in Figure 10b. This plot displayed a good linear relationship between DPV responses and DA concentrations. In addition, a known DA concentration (1 μM) was added to the human serum samples to study the recoveries for repeated three times. The recoveries were obtained in the range of 93.8% to 96.6%, as shown in Table 2. However, the experiment results showed that the slope calculated from related linear regression with the determination of DA in human serum samples was higher than those calculated with the determination of DA in PBS, indicating that some unwanted side effects on the DPV response from the human serum samples and cannot be ignored. Based on this result obtained in this experiment, the proposed Ni-Co-P NSs/Nafion/GCE had a relatively high DPV response for DA determination in human serum samples that may be due to the biocompatibility and the adsorption capacity, which agreed with the observations in previous report [51]. Human serum sample with a suitable dilution using PBS is a commonly used procedure to avoid this unwanted side effects.

## 4. Conclusions

In this study, the successful synthesis of bimetallic nickel/cobalt phosphide nanosheets (Ni-Co-P NSs) exhibited highly interconnected 2D nanosheet structure as well as the synergistic interactions between phosphide and bimetallic Ni/Co atoms, exposing abundant specific surface areas and plentiful available active sites. It could facilitate rapid electron and carrier transfer in order to exhibit a remarkable electrochemical performance toward the electrochemical non-enzymatic DA sensing. The proposed Ni-Co-P NSs showed excellent electrochemical performance (including the linear range from 0.3 to 50 μM, high sensitivity of 2.033 µA µM^−1^ cm^−2^, low limit of detection (LOD) of 0.016 µM, and anti-interference ability). In conclusion, the 2D architectural design of the proposed Ni-Co-P NSs may be a promising potential for practical application in the electrochemical non-enzymatic DA sensing.

## Figures and Tables

**Table 1:** Performance comparison of electrochemical DA sensing based on the different transition meatal (Ni, Co, or Ni/Co) compound materials.

Electrode Materials	Linear Range (μM)	Sensitivity (μA μM^−1^ cm^−2^)	Detection Limit (μM)	Reference
Ag-ZIF-67p/GCE	0.10~100	1.469	0.050	[39]
Ni@CNRs/GCE	0.50~30	0.379	0.056	[43]
Ni-BTC@Ni_3_S_4_/CPE	0.05~750	0.560	0.016	[44]
Ni-MOF/GCE	0.20~100	0.285	0.060	[45]
NiO/NiCo_2_O_4_/CPE	0.10~100	—	0.040	[46]
Ni_2_Co-LDH/GCE	1.30~420	0.148	1.250	[47]
Ni-Co-P NSs/Nafion/GCE	0.3~50	2.033	0.016	This Work

**Table 2:** Real sample analysis.

Added Concentration (μM)	Found Concentration (μM)	Recovery (%)
1	0.966	96.6
1	0.943	94.3
1	0.938	93.8

## Data Availability

The data presented in this study are available on request from the corresponding author. The data are not publicly available due to privacy.
